# Targeting NAD^+^ Metabolism as Interventions for Mitochondrial Disease

**DOI:** 10.1038/s41598-019-39419-4

**Published:** 2019-02-28

**Authors:** Chi Fung Lee, Arianne Caudal, Lauren Abell, G. A. Nagana Gowda, Rong Tian

**Affiliations:** 1 Mitochondria and Metabolism Center, University of Washington, Seattle, WA 98109 USA; 2 Department of Anesthesiology and Pain Medicine, University of Washington, Seattle, WA 98109 USA; 3 Department of Biochemistry, University of Washington, Seattle, WA 98109 USA; 4 Department of Pathology, University of Washington, Seattle, WA 98109 USA; 5 Cardiovascular Biology Research Program, Oklahoma Medical Research Foundation, Oklahoma City, OK 73104 USA

## Abstract

Leigh syndrome is a mitochondrial disease characterized by neurological disorders, metabolic abnormality and premature death. There is no cure for Leigh syndrome; therefore, new therapeutic targets are urgently needed. In Ndufs4-KO mice, a mouse model of Leigh syndrome, we found that Complex I deficiency led to declines in NAD^+^ levels and NAD^+^ redox imbalance. We tested the hypothesis that elevation of NAD^+^ levels would benefit Ndufs4-KO mice. Administration of NAD^+^ precursor, nicotinamide mononucleotide (NMN) extended lifespan of Ndufs4-KO mice and attenuated lactic acidosis. NMN increased lifespan by normalizing NAD^+^ redox imbalance and lowering HIF1a accumulation in Ndufs4-KO skeletal muscle without affecting the brain. NMN up-regulated alpha-ketoglutarate (KG) levels in Ndufs4-KO muscle, a metabolite essential for HIF1a degradation. To test whether supplementation of KG can treat Ndufs4-KO mice, a cell-permeable KG, dimethyl ketoglutarate (DMKG) was administered. DMKG extended lifespan of Ndufs4-KO mice and delayed onset of neurological phenotype. This study identified therapeutic mechanisms that can be targeted pharmacologically to treat Leigh syndrome.

## Introduction

Mitochondrial diseases are caused by mutations in genes that encode mitochondrial proteins. Loss-of-function mutations occur mostly in proteins of the electron transport chain (ETC) or ATP synthase complexes, resulting in defective oxidative phosphorylation^1^. Leigh Syndrome (LS) is a mitochondrial disease caused by wide-spread mutations in ETC proteins, often residing in the mitochondrial Complex I (C-I)^2^. C-I is a large, L-shaped protein complex located in the mitochondrial inner membrane and serves as the primary site for NADH oxidation^3^. C-I contributes to proton gradient generation (mitochondrial membrane potential) across the inner membrane by coupling electron transfer from NADH to other components of the ETC. Mitochondrial membrane potential is utilized to generate ATP and maintain mitochondrial functions. Loss-of-function mutations in C-I in LS leads to inability to oxidize NADH, NAD(H) redox imbalance and impaired ATP production. Therefore, C-I deficiency in LS is devastating to organs with a high energy demand, such as the brain.

Apart from acting as an electron carrier in metabolic pathways, NAD^+^ is a co-substrate of several NAD^+^-dependent enzymes responsible for regulating protein post-translational modifications (PTMs) and DNA repair^4,5^. Mitochondrial function, through regulating NAD(H) redox balance, is critical for the availability of NAD^+^^6–8^. Loss of NAD^+^ induces a pseudohypoxic state and triggers accumulation of HIF1a protein under normoxic conditions^9^. NAD^+^ can be synthesized de novo, or generated by Preiss-Handler and salvage pathways. The salvage pathway is thought to be the most efficient way to replenish NAD^+^^5,10^. Recent studies showed that supplementation of NAD^+^ precursors in the salvage pathway is therapeutic for multiple pathologies^7,11,12^. Importantly, maintaining the NAD^+^ pool by delivering NAD^+^ precursors or inhibition of poly(ADP-ribose) polymerases, treats mitochondrial myopathy and aging by improving mitochondrial function^9,13–15^.

LS manifests an early onset neurodegeneration characterized by progressive losses of mental and motor abilities, retarded growth, lactic acidosis, followed by respiratory failure and premature death^16,17^. A mouse model of LS was developed by the deletion of *Ndufs4* gene (Ndufs4-KO), which encodes a protein necessary for the assembly and stability of C-I. Multiple loss-of-function mutations in *Ndufs4* have been found in LS patients^18,19^. Ndufs4-KO mice show C-I deficiency and recapitulate phenotypes observed in patients with LS^20,21^. We previously showed that C-I-deficiency in the heart of cardiac-specific Ndufs4-KO mice (cKO) led to elevated NADH levels, NAD(H) redox imbalance and protein hyperacetylation^8^. These biochemical changes contribute to the increased susceptibility of the heart to a variety of stresses, which can be rescued by increasing cellular NAD^+^ level.

In this study, we tested the hypothesis that NAD(H) redox imbalance is a pathogenic mechanism in LS using Ndufs4-KO mice. We found that supplementation of the NAD^+^ precursor, nicotinamide mononucleotide (NMN), extended the lifespan of Ndufs4-KO mice. Therapeutic benefits of NMN were mediated by elevation of alpha-ketoglutarate (KG) levels and suppression of hypoxic signaling. Our findings were further supported by the increased lifespan and the neurological benefits of KG supplementation in Ndufs4-KO mice.

## Results

### NMN supplementation extended lifespan of Ndufs4-KO mice

**Figure 1:**
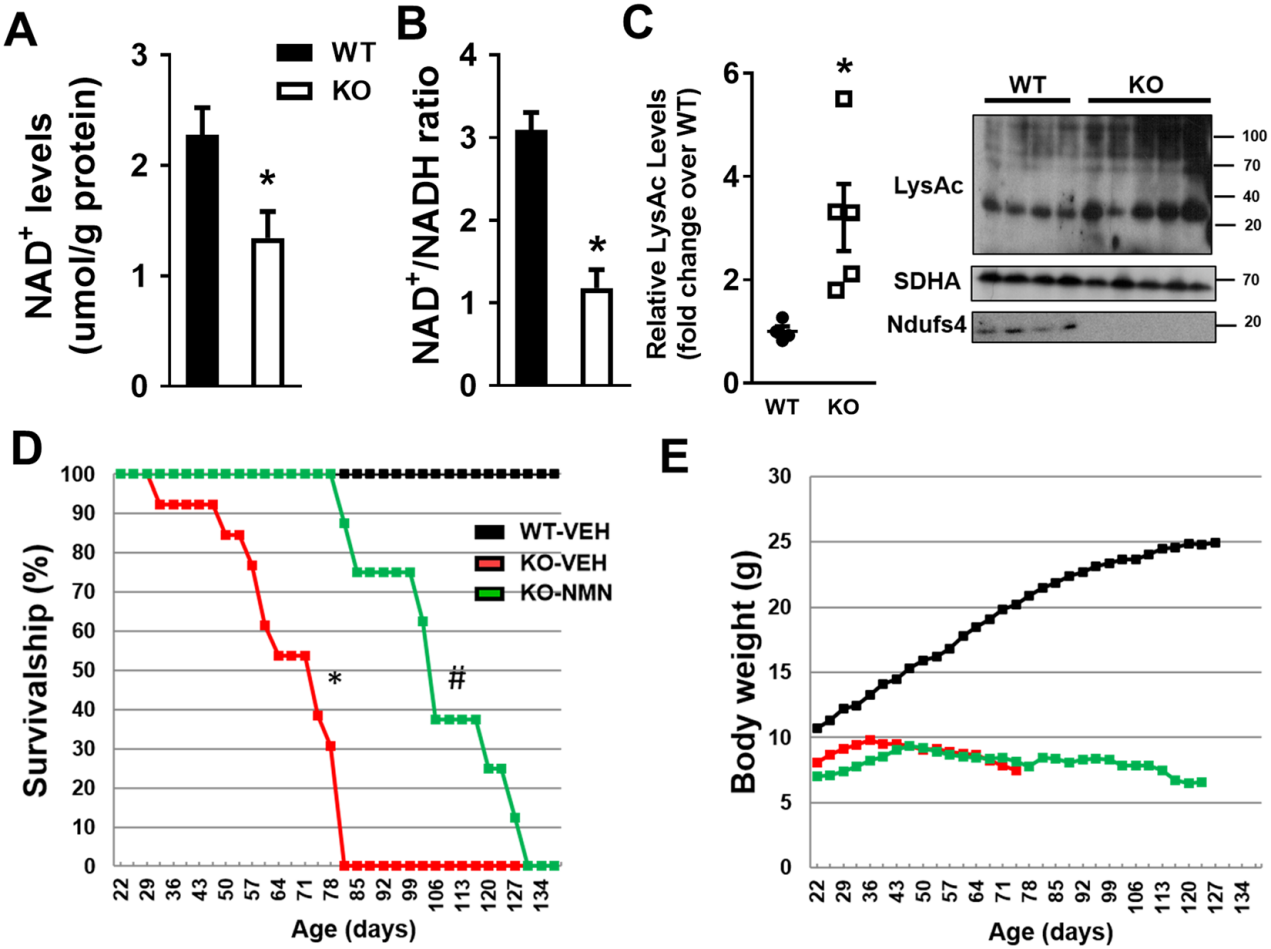
Targeting altered NAD^+^ metabolism of Ndufs4-KO mice to extend lifespan. Brain tissues of age-matched (P-65 to -75) WT and Ndufs4-KO mice were collected. (**A**) NAD^+^ levels (**B**) NAD^+^/NADH ratio and (**C**) protein acetylation (LysAc) levels were measured. SDHA was used as loading control. N = 4–5. Unpaired 2-tailed t-tests were used. (**D**) Survival curves of WT and Ndufs4-KO mice with indicated treatments. N = 10–12. (**E**) Body weight plot of indicated mice. ^*^P < 0.05 versus WT or WT-VEH. ^#^P < 0.05 versus KO-VEH. Log-rank test was used. Full blot images are presented in Supplementary Fig. 7.

We observed a significant decline in NAD^+^ levels and lowered NAD^+^/NADH ratio in the brain of the Ndufs4-KO mice compared to that of age-matched wildtype (WT) mice (Fig. 1A,B, Supplementary Fig. 1). Similar to what was observed in the heart^18,19^, the NAD^+^ redox imbalance was associated with protein hyperacetylation in Ndufs4-KO brain (Fig. 1C). To counteract the decline of NAD^+^ levels in Ndufs4-KO mice, we sought to increase NAD^+^ pool by targeting the NAD^+^ salvage pathway (Supplementary Fig. 2A). We delivered NAD^+^ precursor, nicotinamide mononucleotide (NMN), or vehicle via intraperitoneal injection, to Ndufs4-KO mice starting from postnatal day 21 (P-21) throughout their lifetime (Supplementary Fig. 2B). NMN treatment extended the survival of Ndufs4-KO mice by approximately 2-fold (Fig. 1D; median lifespan 110 days vs 60 days). However, the benefits of NMN appeared to be partial, as the growth of body weight of Ndufs4-KO mice remained stunted after NMN treatment (Fig. 1E).

### Interventions targeting the NAD^+^ salvage pathway failed to impact brain NAD^+^ level of Ndufs4-KO mice

**Figure 2:**
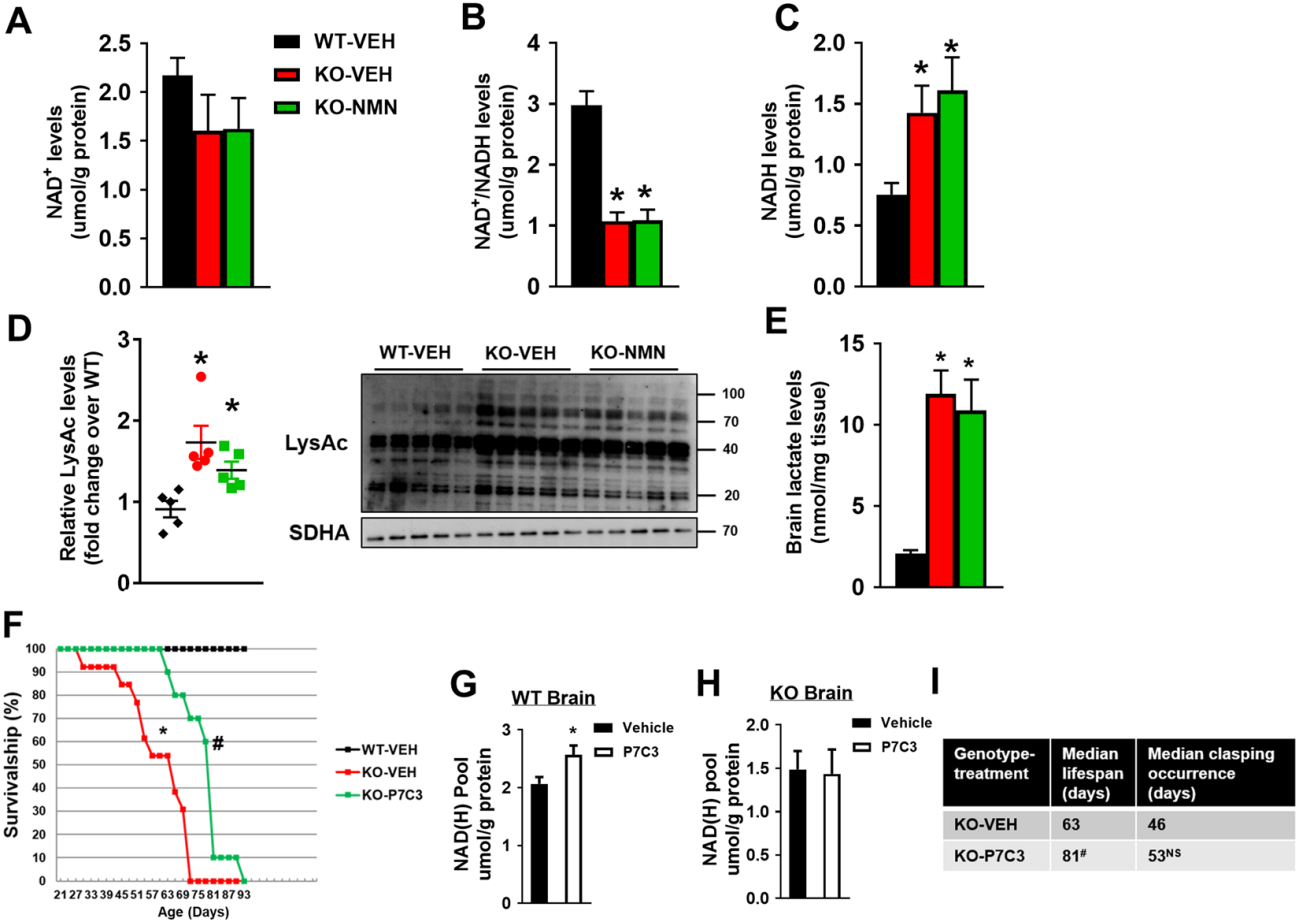
NMN or P7C3 failed to impact NAD^+^ metabolism in Ndufs4-KO brain. Brain tissues of indicated mice were collected at P-50. (**A**) NAD^+^ levels, (**B**) NAD^+^/NADH ratio (**C**) NADH levels of brain from mice as indicated were measured. (**D**) Protein acetylation levels and (**E**) lactate levels in brain from mice as indicated were quantified. N = 5. SDHA was used as loading control. ^*^P < 0.05 versus WT-VEH. One-way ANOVA with Newman-Keuls multiple comparison test was used. Full blot images are presented in Supplementary Fig. 7. WT or Ndufs4-KO mice were treated with P7C3 daily starting from P-21. (**F**) Survival curves of WT and Ndufs4-KO mice treated with VEH or P7C3 were plotted. Log-rank test was used. NAD^+^ pools (NAD^+^ plus NADH levels) of brain tissues from (**G**) WT or (**H**) Ndufs4-KO mice after P7C3 treatment were measured. N = 3. P < 0.05 versus VEH; ^#^P < 0.05 versus KO-VEH. Unpaired 2-tailed t-tests were used. (**I**) Table summarizing median lifespan and clasping occurrence of Ndufs4-KO mice with vehicle or P7C3 treatment. N = 10. ^#^P < 0.05 versus KO-VEH. NS: not statistically significant versus KO-VEH.

Despite prolonged survival, NMN did not elevate NAD^+^ levels or NAD^+^/NADH ratio in the brain of Ndufs4-KO mice harvested at P-50 (Fig. 2A,B, Supplementary Fig. 2C). Levels of NADH and protein acetylation in Ndufs4-KO brains remained high after NMN treatment (Fig. 2C,D). Similarly, protein acetylation levels in heart and liver of Ndufs4-KO mice remained high after NMN treatment (Supplementary Fig. 3A,B). NMN slightly elevated NAD^+^ levels and NAD^+^/NADH ratio in Ndufs4-KO hearts but had no effect in Ndufs4-KO liver (Supplementary Fig. 3C,D). Lactate levels in the brain tissue of Ndufs4-KO mice were elevated and not affected by NMN treatment (Fig. 2E). These data suggested that this regimen did not provide metabolic benefits to Ndufs4-KO brain but may have partial benefits to peripheral tissues. We speculated that this treatment regimen (Supplementary Fig. 2B) did not sufficiently deliver NMN to the brain. To test this hypothesis, we increased NMN dosing frequency to daily injections for seven consecutive days in WT mice (Supplementary Fig. 3E). Although this regimen increased NAD^+^ levels in the heart and liver, it still failed to increase brain NAD^+^ levels (Supplementary Fig. 3F). Using ^1^H NMR spectroscopy^22^ as an alternative method to measure NAD^+^ levels, we still did not observe elevations of NAD^+^ levels in the brain (Supplementary Fig. 3G).

We next sought to stimulate the NAD^+^ salvage pathway by activating its key enzyme, nicotinamide phosphoribosyltransferase (NAMPT), using P7C3^23^ (Supplementary Fig. 2A). P7C3 has been shown to be a proneurogenic agent that protects mice from traumatic brain injury^24^. Daily delivery of P7C3 to Ndufs4-KO mice from P-21 led to a moderate lifespan extension (Fig. 2F, Supplementary Fig. 3H), which was less prominent compared to NMN treatment (median lifespan: 80 vs 110 days). Although P7C3 moderately increased NAD^+^ levels in the brain of WT mice, it failed to do so in the brain of Ndufs4-KO mice (Fig. 2G,H). The inability to elevate NAD^+^ by P7C3 was not due to the lack of NAMPT protein in Ndufs4-KO mice, as NAMPT protein levels did not change (Supplementary Fig. 3I). We also assessed the progression of neurodegeneration using the incidence of forelimb clasping as a neuro-behavior marker^25^. Ndufs4-KO mice showed early clasping phenotype with median occurrence around 46 days as previously observed^25^. P7C3 treatment did not delay the median clasping occurrence in Ndufs4-KO mice (Fig. 2I). These data collectively suggest that it is particularly challenging to boost the NAD^+^ levels in the brain of Ndufs4-KO mice. This prevented us from addressing the efficacy of increasing brain NAD^+^ level as a therapy for LS.

### NMN attenuated NAD^+^ redox imbalance, protein hyperacetylation and suppressed lactate levels of Ndufs4-KO skeletal muscle

**Figure 3:**
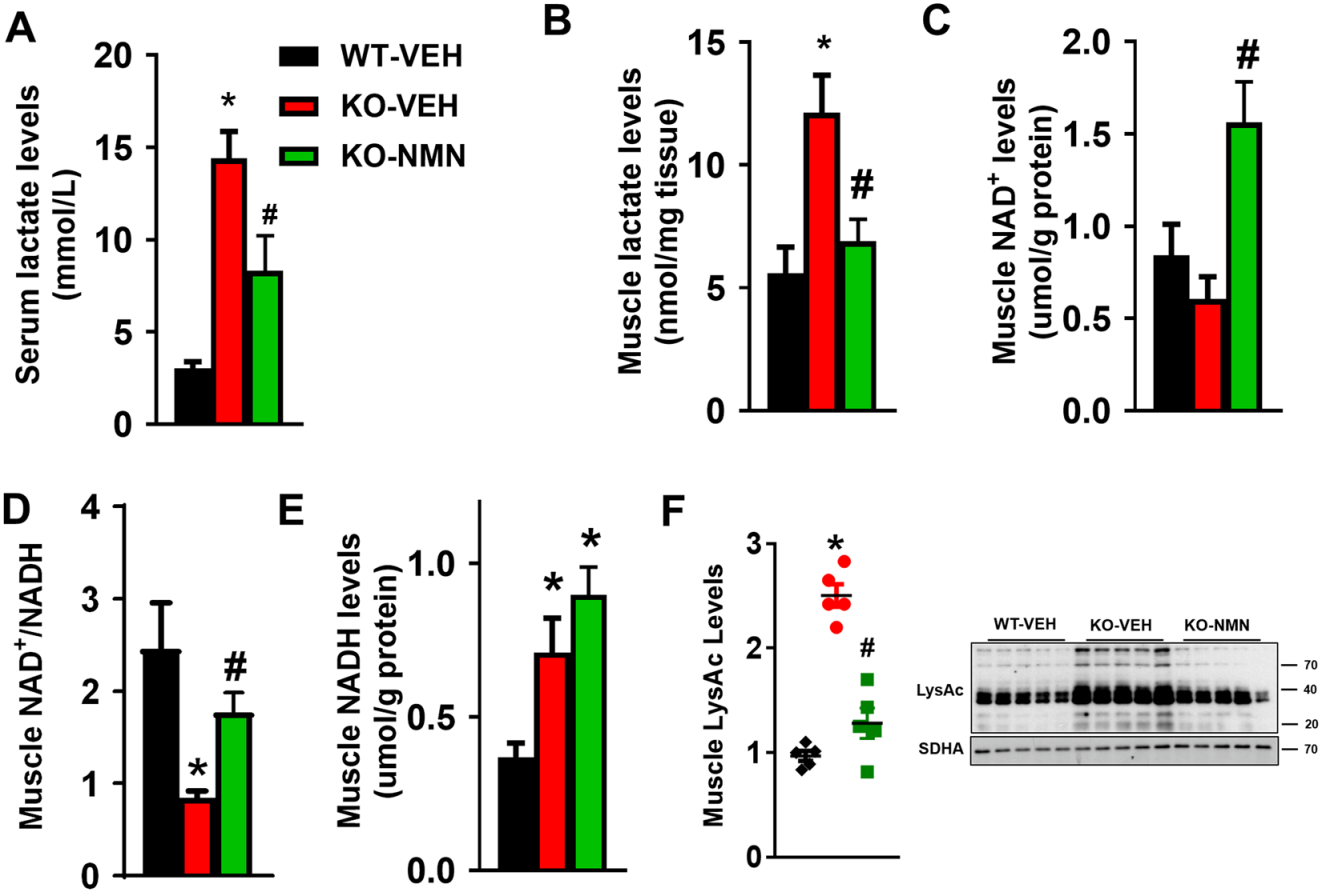
NMN supplementation attenuated NAD^+^ redox imbalance and protein hyperacetylation, and suppressed lactate levels in skeletal muscle of Ndufs4-KO mice. (**A**,**B**) Serum and skeletal muscle of indicated mice were collected at P-50 and lactate levels were measured. (**C**) NAD^+^ levels, (**D**) NAD^+^/NADH ratio and (**E**) NADH levels of skeletal muscle from mice as indicated were measured. (**F**) Protein acetylation levels of skeletal muscle were measured by Western blot. N = 5. SDHA was used as loading control. Full blot images are presented in Supplementary Fig. 7. ^*^P < 0.05 versus WT-VEH; ^#^P < 0.05 versus KO- VEH. One-way ANOVA with Newman-Keuls multiple comparison test was used.

We next examined the non-neurological impacts of NMN supplementation on Ndufs4-KO mice to account for the lifespan extension (Fig. 1D). As in LS patients, serum lactate levels were elevated in Ndufs4-KO mice compared to WT mice (Fig. 3A). NMN treatment suppressed serum lactic acidosis in Ndufs4-KO mice (Fig. 3A), suggesting an improvement of systemic metabolism by the treatment. NMN suppressed lactate levels in skeletal and cardiac muscles of Ndufs4-KO mice (Fig. 3B, Supplementary Fig. 4A). In parallel to the attenuated lactic acidosis, the NAD^+^ pool was expanded in the skeletal muscle and the heart (Fig. 3C and Supplementary Fig. 3C). NAD^+^ redox balance in skeletal muscle and heart of Ndufs4-KO mice was partially restored by NMN despite a higher level of NADH (Fig. 3C–E, Supplementary Fig. 3C). Normalization of tissue NAD^+^/NADH ratio would reduce the conversion of pyruvate to lactate by lactate dehydrogenase (LDHA), contributing to the lowered tissue and serum lactate levels (Fig. 3B). Attenuated NAD^+^ redox imbalance by NMN lowered protein hyperacetylation in skeletal muscle of Ndufs4-KO mice (Fig. 3F). NMN treatment also improved cardiac function, as measured by murine echocardiography, in Ndufs4-KO mice (Supplementary Fig. 4B). These results suggest that the lifespan extension of Ndufs4-KO mice by NMN is attributable to improved metabolism and function in peripheral tissues, independent of the brain.

### NMN blunted activation of hypoxic signaling in Ndufs4-KO mice

**Figure 4:**
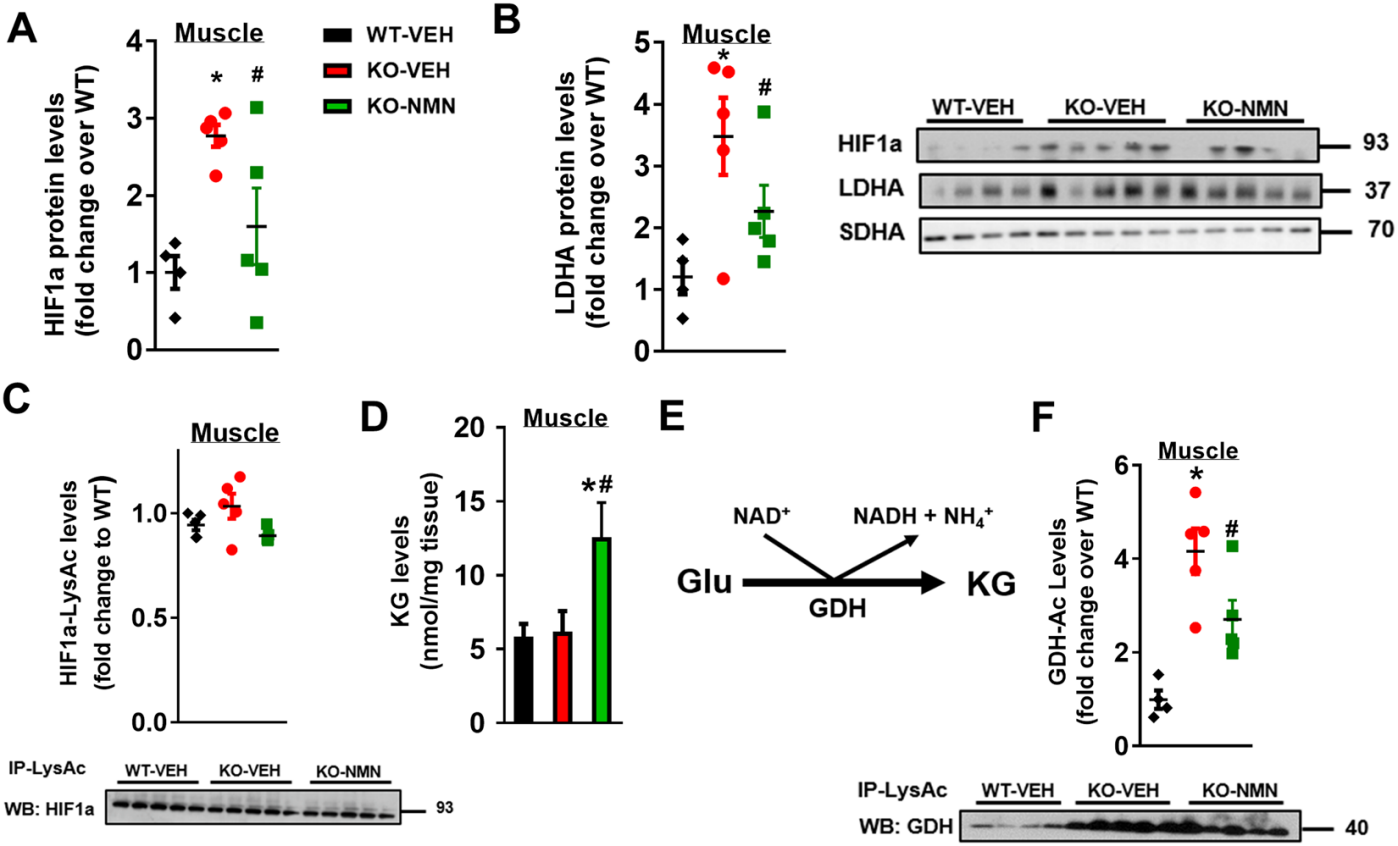
NMN blunted the activation of hypoxia signaling in Ndufs4-KO muscle via up-regulation of α-ketoglutarate (KG) levels. Protein levels of (**A**) HIF1a and (**B**) LDHA of skeletal muscle from mice as indicated were measured by Western blot. SDHA was used as loading control. (**C**) Acetylation levels of HIF1a and (**D**) KG levels from skeletal muscle of mice as indicated were quantified. (**E**) Glutamate dehydrogenase (GDH) catalytic reaction. (**F**) Acetylation levels of GDH in skeletal muscle from mice treated as indicated were measured by Western blot analysis. N = 4–5. ^*^P < 0.05 versus WT-VEH; ^#^P < 0.05 versus KO-VEH. One-way ANOVA with Newman-Keuls multiple comparison test was used. Full blot images are presented in Supplementary Fig. 7.

Defective oxidative metabolism in mitochondrial disease promotes anaerobic glycolysis that produces lactate from pyruvate while regenerating NAD^+^ from NADH. Sustained glycolysis eventually lowers NAD^+^/NADH ratio. The resultant lactic acidosis and abnormal NAD^+^ redox state are markers in LS and linked to cardiovascular risk and neurodegeneration^26^. We found that hypoxia inducible factor 1 alpha (HIF1a), an important transcriptional activator of glycolysis, was elevated in skeletal muscle and brain of Ndufs4-KO mice (Fig. 4A, Supplementary Fig. 5A). The HIF1a downstream protein targets, such as LDHA, were also increased in Ndufs4-KO skeletal muscle and brain (Fig. 4B, Supplementary Fig. 5A). HIF1a can be stabilized by ROS or protein acetylation^27–29^. Although protein acetylation levels of Ndufs4-KO skeletal muscle and brain were elevated (Figs 2D and 3F), we did not detect changes in the acetylation level of HIF1a protein (Fig. 4C, Supplementary Fig. 5B). Instead, hyperacetylation of a key mitochondrial antioxidant enzyme, superoxide dismutase 2 (SOD2), was found in the brain of Ndufs4-KO mice (Supplementary Fig. 5C). Acetylation of SOD2 has been shown to inhibit its ROS scavenging activity^30^. To determine whether a higher level of ROS contributed to HIF1a accumulation in Ndufs4-KO mice, protein nitrotyrosine (NT) levels, a marker of oxidative stress, were measured. Protein nitrotyrosine levels were elevated in Ndufs4-KO brain (Supplementary Fig. 5D), consistent with a prior report of increased ROS accumulation in the brain of Ndufs4-KO mice^31^. These results collectively suggest that ROS-induced HIF1a accumulation is a likely cause of increased glycolysis in Ndufs4-KO brain.

NMN supplementation lowered levels of HIF1a and LDHA proteins in the skeletal muscle but not in the brain of Ndufs4-KO mice (Fig. 4A,B, Supplementary Fig. 5A). This finding is coincident with the expanded NAD^+^ pool and the lowered lactate levels in skeletal muscle of Ndufs4-KO mice by NMN treatment, but not in the brain (Figs 2–3). These results collectively suggest that hypoxic signaling and glycolysis in Ndufs4-KO mice could be manipulated via NAD^+^-sensitive mechanisms. We found that alpha-ketoglutarate (KG) levels were elevated in Ndufs4-KO muscle after NMN treatment (Fig. 4D). As KG is a required co-substrate for hydroxylation of HIF1a for degradation, increased KG promotes HIF1a reduction. KG can be produced from glutamate via glutamate dehydrogenase (GDH) reaction, which is coupled with conversion of NAD^+^ to NADH in the direction of KG production (Fig. 4E). Since NMN elevated NAD^+^/NADH ratio in Ndufs4-KO skeletal muscle (Fig. 3D), KG production was favored. Furthermore, hyperacetylation of GDH causes a loss of catalytic activity due to conformational change^32,33^. NMN treatment lowered GDH hyperacetylation in Ndufs4-KO skeletal muscle but not in brain (Fig. 4F, Supplementary Fig. 5E). These data suggest that increasing NAD^+^ level enhances KG production (Fig. 6), which in turn attenuates hypoxic signaling in Ndufs4-KO mice.

### DMKG supplementation increased lifespan, improved neurological phenotype and suppressed hypoxic signaling of Ndufs4-KO mice

**Figure 5:**
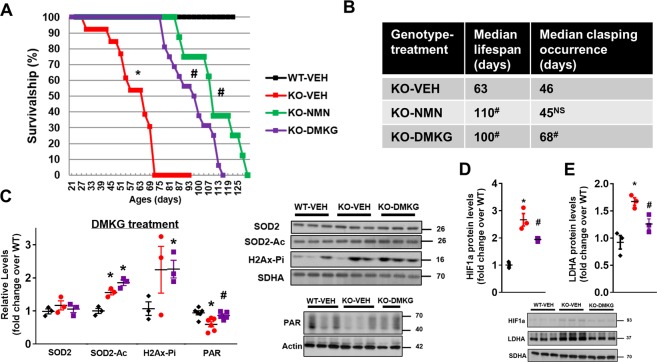
Supplementation of dimethyl α-ketoglutarate (DMKG) extended lifespan and delays the onset of clasping in Ndufs4-KO mice. (**A**) Survival curves of WT mice, KO mice treated with vehicle (VEH), NMN or DMKG. N = 10–16. (**B**) Table summarizing median lifespan and clasping occurrence of Ndufs4-KO mice with vehicle, NMN or DMKG treatments. N = 10–16. Log-rank test was used. (**C**) Levels of SOD2 protein, SOD2 acetylation, H2Ax phosphorylation (H2Ax-Pi), and protein PAR in brain tissues in DMKG treatment cohort at P-50 were quantified. N = 3–6. Protein levels of (**D**) HIF1a and (**E**) LDHA in brain tissues were measured by Western blots. N = 3. Full blot images are presented in Supplementary Fig. 7. ^*^P < 0.05 versus WT-VEH; ^#^P < 0.05 versus KO-VEH. NS: not statistically significant versus KO-VEH. One-way ANOVA with Newman-Keuls multiple comparison test was used. SDHA and actin were used as loading control.

We next sought to directly increase KG level in the brain of Ndufs4-KO mice using dimethyl-ketoglutarate (DMKG), a cell permeable form of KG. DMKG was administered to Ndufs4-KO mice intraperitoneally, daily starting from P-21 (Supplementary Fig. 6A). We observed a significant lifespan extension in DMKG-treated Ndufs4-KO mice compared to vehicle-treated Ndufs4-KO mice (Fig. 5A,B). The median lifespan of DMKG-treated Ndufs4-KO mice was similar to NMN-treated mice (100 days in DMKG-treated Ndufs4-KO mice versus 110 days in NMN-treated group).

Using positive forelimb clasping as a readout^25^, we assessed and compared the effects of NMN and DMKG on the neurological system of Ndufs4-KO mice. It has been shown that neurological symptoms in Ndufs4-KO mice manifested around P-35, which coincided with the body weight peak of the mice (Supplementary Figs 1E and 6B) as previously shown^20,25^. Survivorship of Ndufs4-KO mice declined after P-35 as the incidence of clasping progressively increased (Fig. 5A, Supplementary Fig. 6B). NMN treatment did not change the incidence of clasping of Ndufs4-KO mice (median clasping occurrence of P-45 versus P-46 in vehicle, Fig. 5B, Supplementary Fig. 6B) as the brain NAD^+^ pool was not expanded. In contrast, DMKG treatment delayed incidence of clasping in Ndufs4-KO mice with a median clasping occurrence at P-68 (Fig. 5B, Supplementary Fig. 6B). Consistent with the unchanged NAD^+^ levels in brain of Ndufs4-KO mice (Fig. 2A), NMN treatment did not alter hyperacetylation of SOD2 in Ndufs4-KO brain, and DNA damage, as evidenced by elevated histone 2 A phosphorylation (H2Ax-Pi) remained high (Supplementary Figs 5 and 6C). Although NMN lowered acetylation levels of SOD2 in Ndufs4-KO muscle, protein nitrotyrosine levels (NT) were not altered in Ndufs4-KO muscle or by NMN treatment (Supplementary Fig. 6D). These data suggested that oxidative stress contributed to the impairments of Ndufs4-KO brain, but not in skeletal muscle, and the benefits of NMN treatment were not mediated by relieving oxidative stress. DMKG treatment did not alter SOD2 acetylation and histone 2A phosphorylation in Ndufs4-KO brain (Fig. 5C). However, DMKG elevated protein poly-ADP-ribosylation levels in Ndufs4-KO brain (Fig. 5C, Supplementary Fig. 6E). Importantly, DMKG treatment suppressed hypoxia signaling by lowering HIF1a and LDHA levels in Ndufs4-KO brain without affecting the NAD^+^ pool of Ndufs4-KO brain (Fig. 5D,E, Supplementary Fig. 6F). These results suggest that DMKG is a viable option to suppress hypoxia signaling independent of the NAD^+^ salvage pathway in mitochondrial disease.

## Discussion

**Figure 6:**
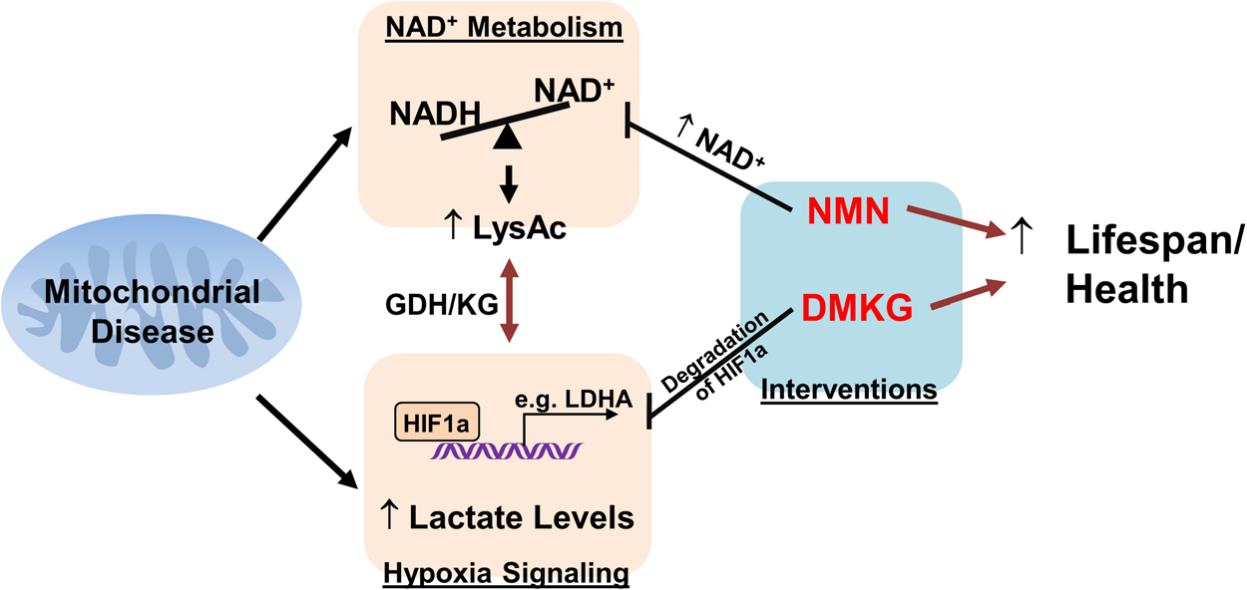
Targeting NAD^+^ metabolism or hypoxia signaling as interventions for Leigh Syndrome, a mitochondrial disease. Complex I deficiency in Ndufs4-KO mice triggers NAD^+^ redox imbalance, activation of hypoxia signaling and lactate acidosis. NMN and DMKG treatment can improve lifespan and health of Ndufs4-KO mice via normalization of these pathways.

This study identifies two potential therapeutic targets to treat mitochondrial diseases, namely NAD^+^ metabolism and hypoxia signaling (Fig. 6). Interventions targeting these two pathways attenuate disease progression and extend lifespan in a mouse model of Leigh Syndrome. Supplementation of a NAD^+^ precursor, NMN, restores NAD^+^ redox balance and protein acetylation in muscle and attenuates lactate acidosis. These benefits are achieved by suppressing the activation of hypoxia signaling and elevations of glycolytic proteins in the muscle. A similar effect can be achieved by boosting intracellular KG levels to promote HIF1a degradation. These findings are of translational significance as compounds for increasing intracellular NAD^+^ or suppressing HIF1a activation are becoming available for clinical use^7,34,35^.

Supplementation of NMN showed the most remarkable effects on lifespan extension of Ndufs4-KO mice. However, the NAD^+^ pool and NAD^+^-dependent biochemical pathways in the brain of Ndufs4-KO mice were not responsive to NMN treatment. Consequently, we did not observe any improvement of neurological outcomes either *in vivo* or at cellular levels. The ineffectiveness to elevate brain NAD^+^ pool by NMN is likely multifactorial. One possibility is that NMN does not reach the brain. However, a recent study showed that NMN attenuated ischemia injuries and protected the blood-brain barrier^11^, suggesting that NMN can improve brain function. Another possibility is that supplementation of NAD^+^ precursor increases turnover but not the size of the NAD^+^ pool. Early studies indicated that the half-life of the NAD^+^ pool was as short as 6–10 hours^36,37^ and the quick turnover was likely due to robust activities of NAD^+^-consuming enzymes such as Sirtuins. In our study, NMN treatment did not change protein acetylation levels in the brain of Ndufs4-KO mice. Therefore, rapid NAD^+^ consumption by Sirtuin deacetylases is unlikely the reason for the stagnant NAD^+^ pool in the brain of Ndufs4-KO mice during NMN treatment. Gauging activities of Sirtuins with total acetylation levels is not without limitation, as multiple Sirtuins exist in different sub-cellular locations and Sirtuins regulate post-translational modifications other than acetylation. Therefore, we employed another pharmacologic approach that elevates NAD^+^ pool to test our hypothesis. Targeting the NAD^+^ salvage pathway by P7C3, a putative NAMPT activator, increased the brain NAD^+^ pool in WT mice but not in Ndufs4-KO mice at the same dose. The results suggested that impaired mitochondrial function in Ndufs4-KO mice could be responsible for the failure to expand the NAD^+^ pool. However, we cannot rule out any non-specific effects of P7C3 or that a higher dose is required to reverse the molecular and physiological deficits in Ndufs4-KO mice. Generation of NAD^+^ from NAD^+^ precursors requires high energy phosphate compounds such as ATP and phosphoribosyl pyrophosphate (PRPP) as substrates^38^. Complex I-deficiency in Ndufs4-KO results in severe energy deprivation in the brain and liver whereas muscle and heart are largely spared^8,20,39^. The organ-selective change in energy status in Ndufs4-KO mice matches the effectiveness of raising the intracellular NAD^+^ level in the present study.

Supplementation of DMKG, a KG precursor, demonstrates neurological benefit in the present study. Besides the suppression of hypoxic signaling observed here, KG was found to support mitochondrial bioenergetics in Ndufs4-KO^40^. Contrary to the loss of Complex I supported respiration, KG-driven respiration is unaffected in the mitochondria isolated from the brain of Ndufs4-KO mice, and it is even elevated in older Ndufs4-KO mice^40^. Thus, KG may be used by Ndufs4-KO mitochondria as an alternative substrate for Complex-II dependent respiration, which compensates for the deficiency of Complex-I function. It is noteworthy that elevation of KG levels has been associated with improved outcome in Ndufs4-KO mice treated by rapamycin in a recent study^25^ as well as by NMN in the present study. KG inhibits the TOR pathway and PARP1 is regulated by protein phosphorylation^41^. Thus, we speculate that DMKG supplementation increases PAR levels in Ndufs4-KO brain by suppression of PARP1 activity via inhibition of mTOR, which is consistent with the benefit of rapamycin treatment in Ndufs4-KO mice. These findings suggest that KG level is likely a therapeutic target and a readout for treatment efficacy in Leigh syndrome. Further studies are needed to define the therapeutic mechanisms of DMKG.

Mitochondrial dysfunction is known to activate hypoxia signaling under normoxic conditions. Decline in NAD^+^/NADH ratio, also known as pseudohypoxic state, plays an important role in mitochondria-nuclear communication in aging and diseases^9^. Results from the present study suggest that correction of pseudohypoxic status is therapeutic for mitochondrial disease. Interestingly, a recent study showed that the opposite strategy, i.e. subjecting Ndufs4-KO mice to chronic hypoxia environment, is also therapeutic^42^. These seemingly contradictory observations suggest that metabolic remodeling in organisms with mitochondrial defect is in discordant with the oxygenated environment. Blocking the aberrant remodeling, such as protein hyperacetylation and HIF1a activation, prevents the incongruity. Likewise, providing a hypoxic environment would accommodate the upregulation of hypoxia metabolism and avoid oxygen toxicity^35,42^.

As discussed above, several treatment strategies have improved survival of Ndufs4-KO mice, e.g. rapamycin, chronic hypoxia, NAD^+^ precursor (NMN) and DMKG. Although the effect of lifespan extension by supplementation of NAD^+^ precursor is not as dramatic as hypoxic chamber and rapamycin treatments, this strategy has a high potential for translation. Several NAD^+^ precursors are available as nutraceuticals. They are safe and well tolerated in humans even at high doses^43^. In a previous study, rapamycin treatment started on P-21 at a dose comparable to clinical use resulted in a similar lifespan extension in Ndufs4-KO as the NMN treatment described here. However, increasing the rapamycin dose several folds higher and starting the treatment from P-10 drastically improve the efficacy^25^. In the present study, all treatments started from P-21. It remains to be determined whether a higher dose and/or earlier treatment will yield greater improvement. As restoration of the brain NAD^+^ pool is likely energy-dependent, early intervention before severe energy depletion is desirable. Thus, the optimal time to initiate interventions is worth further investigation for maximum benefit.

## Materials and Methods

### Animals care and experiments

All animal care and procedures were approved by the Institutional Animal Care and Use Committee (IACUC) at the University of Washington and were performed in accordance with IACUC guidelines and regulations. Ndufs4 heterozygous deletion (Ndufs4^+/−^) mice were generated from breeding of Ndufs4^flox/flox^ mice (Palmiter laboratory) with Meox2-Cre expressing mice (Jackson laboratory). Breeding of Ndufs4^+/−^ male and female mice generated Ndufs4^−/−^ mice (KO). Mice were weaned at 21 days of age (P-21). KO mice were housed with control littermates (Ndufs4^+/−^ or Ndufs4^+/+^) in the same cage. These mice were genotyped at P-21. Both male and female mice were used in the study. The survival curves were analyzed by pooling the data from both sexes (male-to-female ratio is ~1:1), as there was no sex-specific difference in survivalship. Survival of KO mice was recorded. KO mice were euthanized after veterinarian consultation if 20% loss in maximum body weight, severe dehydration and immobility were observed. Body weight and incidence of clasping^25^ were monitored starting from P-21 until death. Forelimb clasping is a marker of the progression of neurodegeneration^25^. WT or KO mice were grasped with their tails and lifted clear of surrounding objects. Forelimb position was observed. If forelimb retraction to the midline of the body is observed, it will be scored positive for clasping^25^. Tissue harvest was performed in the afternoon during light cycle without fasting.

Nicotinamide mononucleotide (NMN, N3501-Sigma) was dissolved in sterile saline at 50 mg/ml and delivered intraperitoneally to mice at a dose of 500 mg/kg/day twice a week. P7C3 (4076-Tocris) was dissolved in 10% Cremopho EL at 5 mg/ml and delivered intraperitoneally to mice at dose of 50 mg/kg/day daily. Dimethyl-a-ketoglutarate (DMKG, 349631-Sigma) was dissolved at 30 mg/ml in sterile saline and delivered intraperitoneally to mice at dose of 300 mg/kg/day daily.

Cardiac function was assessed by echocardiography using VEVO 2100 system (VisualSonics) on lightly-anesthetized mice. Measurements were made when heart rate was within 500–600 beats per minute. Cardiac function was measured in parasternal long axis B- and M-mode images and calculated by average of three cardiac cycles. Fractional shortening (FS) was calculated using ultrasound analysis software for image data associated with Vevo 2100 (VisualSonics).

### Western blotting

Blood inside organs were removed by rinsing tissues with PBS. Tissues were snap-frozen under liquid nitrogen. Tissues were homogenized in RIPA buffer (Sigma) with protease (Roche), deacetylase (nicotinamide, Tricostatin A) and phosphatase inhibitors (Roche)^7^. Protein concentrations of samples were determined by Lowry assay and each amount of protein (30–50 ug per sample) were loaded for SDS-PAGE. Antibodies from the following companies were used for Western blot analysis: Phospho-H2Ax (1:500, NBP1-19255-Novus Biological), PAR (1:500, AM80100UG-Millipore), actin (1:5000, sc-8432-Santa Cruz), nitrotyrosine (1:500, 06-284-Millipore) acetyl-lysine (1:1000, 9441-Cell signaling), HIF1a (1:400, 14179-Cell Signaling), GLUT1 (1:500, ab652-Abcam), PDK4 (1:1000, 3820-Cell Signaling), LDHA (1:1000, 3582-Cell Signaling), SDHA (1:10000, ab14715-Abcam), Ndufs4 (1:1000, ab87399-Abcam,), SOD2 (1:1000, ab16956-Abcam) SOD2-Ac (1:1000, ab137037-Abcam), GDH (1:1000, 12793-Cell Signaling). Blots were blocked in 5% BSA-TBST. Antibodies were diluted in 5% BSA-TBST. Protein bands were visualized with chemiluminescence assay (Pierce) with secondary antibodies coupled with HRP. The protein abundance was analyzed by densitometry with ImageJ. SDHA or actin were used as a loading control for quantification.

### Detections of metabolites

Assay kits were used to measure levels of NADH and NAD^+^ (BioAssay, CA, USA), alpha-ketoglutarate (Sigma, MO, USA), serum and tissue lactate (Trinity Biotech, Ireland) by following manufacturers’ instructions. Tissues for metabolite measurements were collected by snap-freezing.

For 1H NMR spectroscopy, methanol, chloroform, monosodium phosphate (NaH_2_PO_4_), disodium phosphate (Na_2_HPO_4_), sodium salt of 3-(trimethylsilyl)propionic acid-2,2,3,3-d_4_ (TSP) were obtained (Sigma, MO, USA). Deuterium oxide (D_2_O) was obtained from Cambridge Isotope laboratories, Inc. (Andover, MA).

To extract mouse heart tissues by methanol and chloroform extractions, weighed tissue specimens were mixed with a 1 mL mixture of cold methanol and chloroform (1:2 v/v; 4 °C) in 2 mL Eppendorf vials, homogenized and sonicated for 20 s. A further 800 µL (cold chloroform/distilled water mixture (1:1 v/v) was added, the sample was then vortexed and set aside for 30 min on ice to separate the solvent layers. Next, after centrifugation at 2039 rcf, the aqueous (top) layer was separated and filtered using 1.5 mL 0.2 mm syringe filters and freeze dried. The dried extracts were mixed with 220 µL of a cold phosphate buffer (0.1 M; pH = 7.45; 4 °C) in D_2_O containing 50 µM TSP and the solutions were transferred to 3 mm sample tubes for measurements. The phosphate buffer was degaussed by bubbling helium gas for 30 min prior to preparation of the samples for NMR analysis.

All NMR experiments were performed at 298 K. A Bruker Avance III 800 MHz spectrometer equipped with a cryogenically cooled probe and *z*-gradients suitable for inverse detection was used. One dimensional NOESY pulse sequence with residual water suppression using pre-saturation, 10204 Hz spectral width, 6.6 s total recycle delay, 64 transients and 32 K time domain points were used for ^1^H 1D NMR experiments. Chemical shifts were referenced to the internal TSP signal. Bruker Topspin versions 3.0 or 3.1 software packages were used for NMR data acquisition, processing, and analyses.

Identification of the coenzyme peaks in the NMR spectra was based on the database and peak assignments established recently^22^. The Chenomx NMR Suite Professional Software package (version 5.1; Chenomx Inc., Edmonton, Alberta, Canada) was used to quantify the coenzyme peaks. Peak fitting with reference to the internal TSP signal enabled the determination of absolute concentrations of the coenzymes. Concentrations of the coenzymes were expressed both relative to the internal reference TSP as well as the internal taurine.

### Statistical analysis

Comparisons among the multiple groups were performed by 1-way ANOVA, followed by Newman-Keuls multiple comparison test. For comparisons involving only two groups, unpaired 2-tailed t-tests were used. Statistical comparisons of survival curves and clasping were performed using log-rank test. All analyses were performed using GraphPad Prism 7.0. All data are expressed as mean ± SEM and a *p* < 0.05 was considered significant.

## Supplementary information

Supplemental materials

## Data Availability

The data that support the findings of this study are included in this manuscript and available from corresponding author upon request.
