# Supplementary material for: Targeting NAD+ Metabolism as Interventions for Mitochondrial Disease
Source: Sci Rep. 2019 Feb 28;9:3073. doi: 10.1038/s41598-019-39419-4 (PMC6395802; doi:10.1038/s41598-019-39419-4)
Supplement: Supplementary file 1 — Supplemental materials [file 41598_2019_39419_MOESM1_ESM.docx]

**Targeting NAD^+^ Metabolism as Interventions for Mitochondrial Disease**

^1,2^Chi Fung Lee, ^1,3^Arianne Caudal, ^1,4^Lauren Abell, ^1,2^Nagana Gowda, ^1,2,3,4^Rong Tian*

^1^Mitochondria and Metabolism Center, ^2^Department of Anesthesiology and Pain Medicine, ^3^Department of Biochemistry, ^4^Department of Pathology, University of Washington, Seattle, WA 98109, USA

**Supplementary Information**


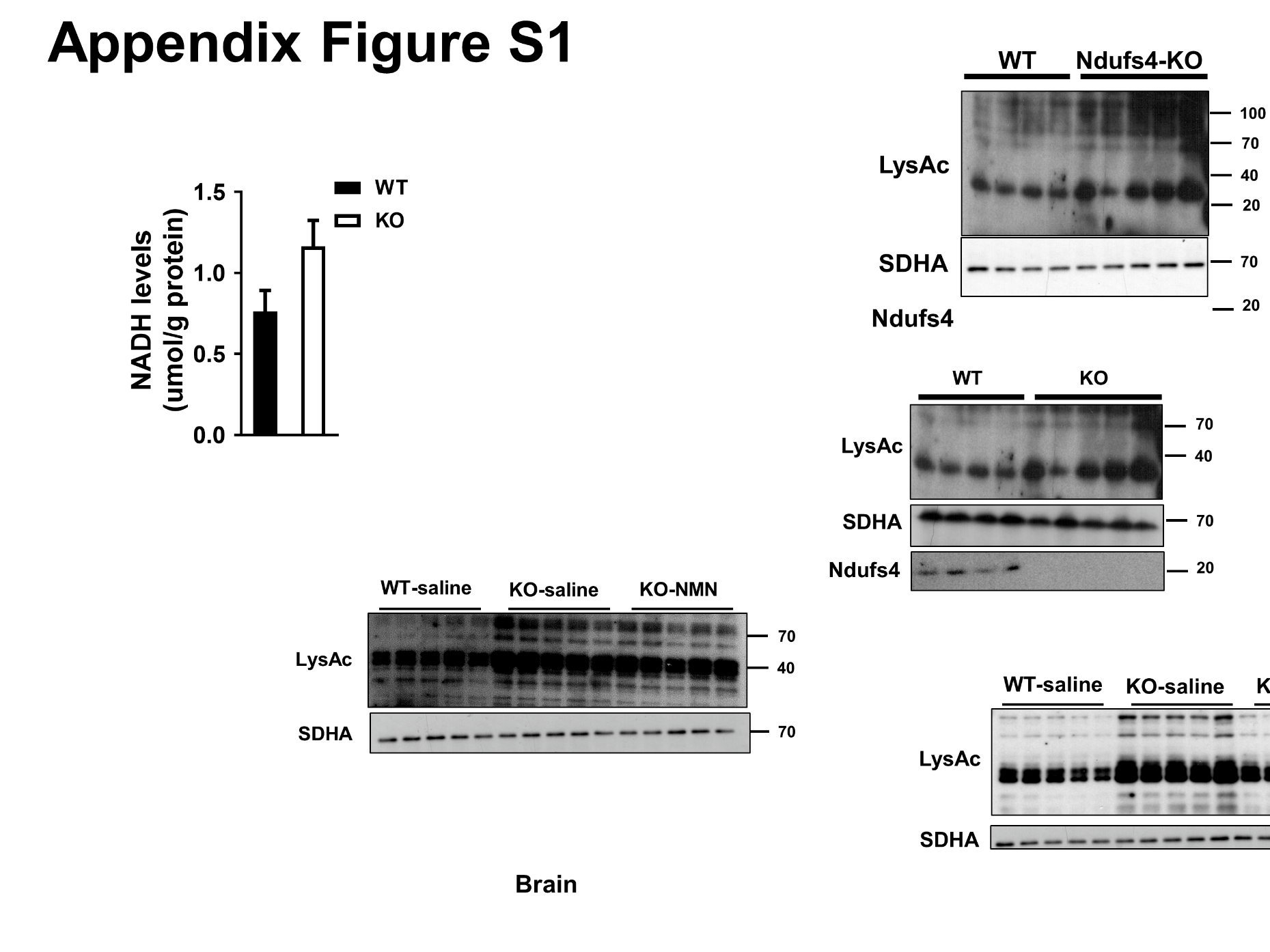


Supplementary figure 1. NADH levels of WT and KO brain tissues were measured by biochemical assay kit.


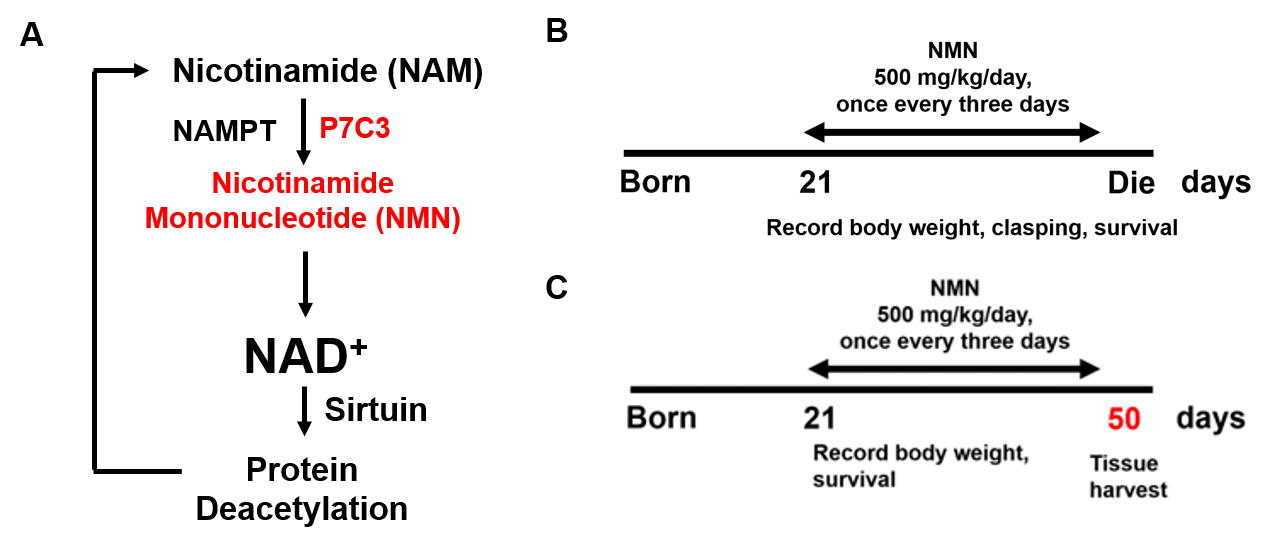
Supplementary figure 2. (A) The NAD^+^ salvage and sirtuin-dependent deacetylation pathways. NMN and P7C3 are the compounds tested to activate the NAD^+^ salvage pathway for elevating NAD^+^ levels. (B) Treatment regimen of NMN for the survival study of WT and Ndufs4-KO mice. (C) Treatment regimen of NMN for biochemical analyses of tissues collected from WT and Ndufs4-KO mice after treatment.

**
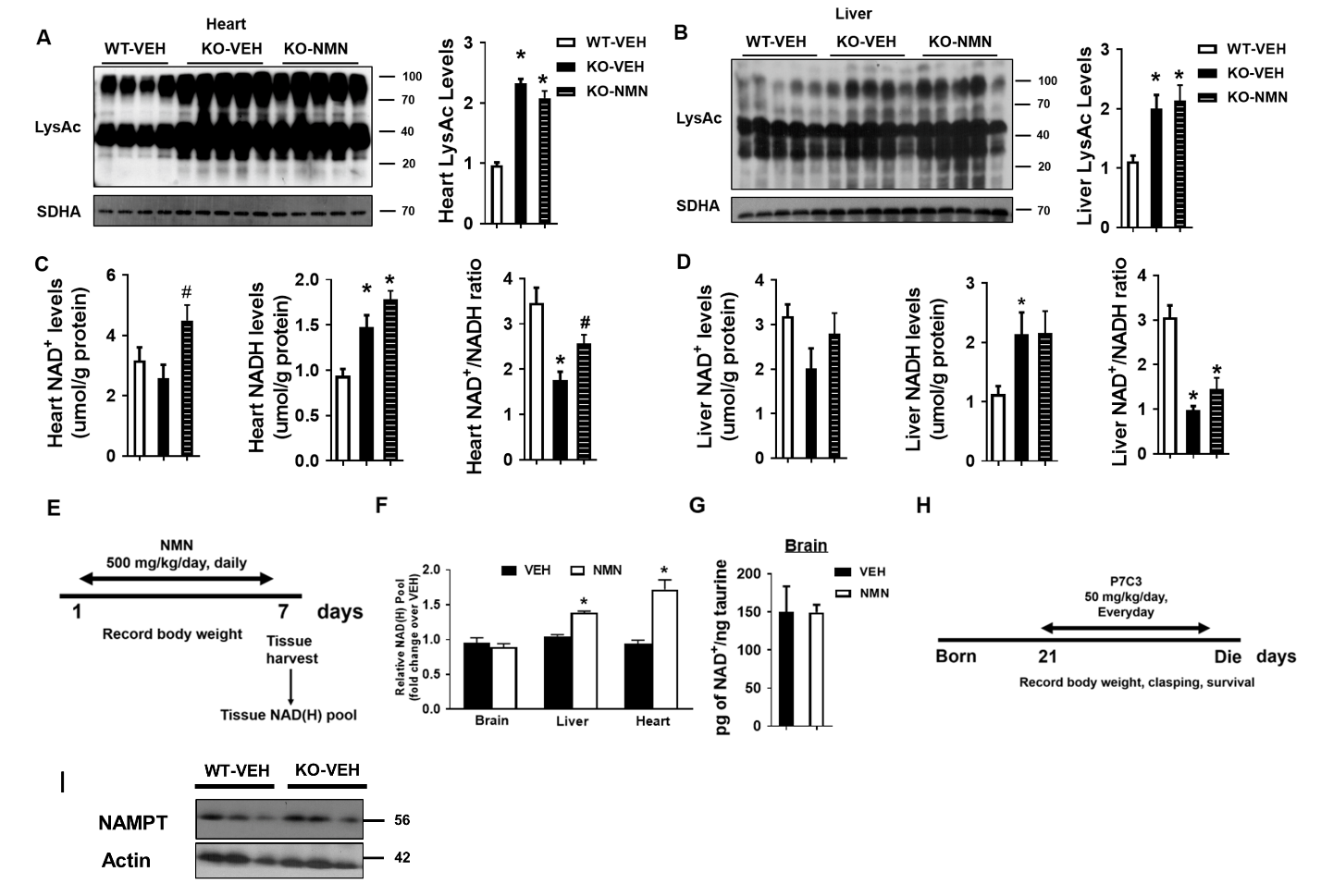
**

Supplementary figure 3. Protein acetylation levels of (A) heart and (B) liver tissues from indicated mice were quantified by Western blots. SDHA was used as loading control. Levels of NAD^+^, NADH, and NAD^+^/NADH ratio of (C) heart and (D) liver tissues from indicated mice were quantified. *: P<0.05 versus WT-VEH. #: P<0.05 versus KO-VEH. One-way ANOVA was used. (E) Treatment plan for the daily NMN experiment. (F) NAD(H) pools of brain, liver, and heart tissues were measured by biochemical assay kits. (G) NAD^+^ levels of brain tissues were measured by 1H NMR spectroscopy. *: P<0.05 versus VEH. Unpaired 2-tailed t-tests were used. (H) Treatment plan for the daily P7C3 experiment. (I) Levels of NAMPT in brain of indicated mice were measured by Western blot. Actin was used as loading control.


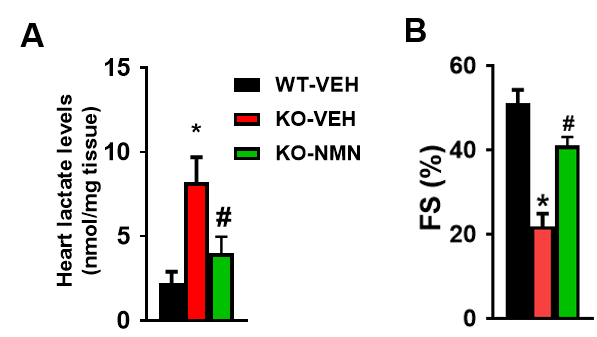


Supplementary figure 4. (A) Cardiac muscle of indicated mice were collected and lactate levels were measured. (B) Cardiac function, reported as fractional shortening, of indicated mice were measured by echocardiography *: P<0.05 versus WT-VEH; #: P<0.05 versus KO-VEH. One-way ANOVA with Newman-Keuls multiple comparison test was used.


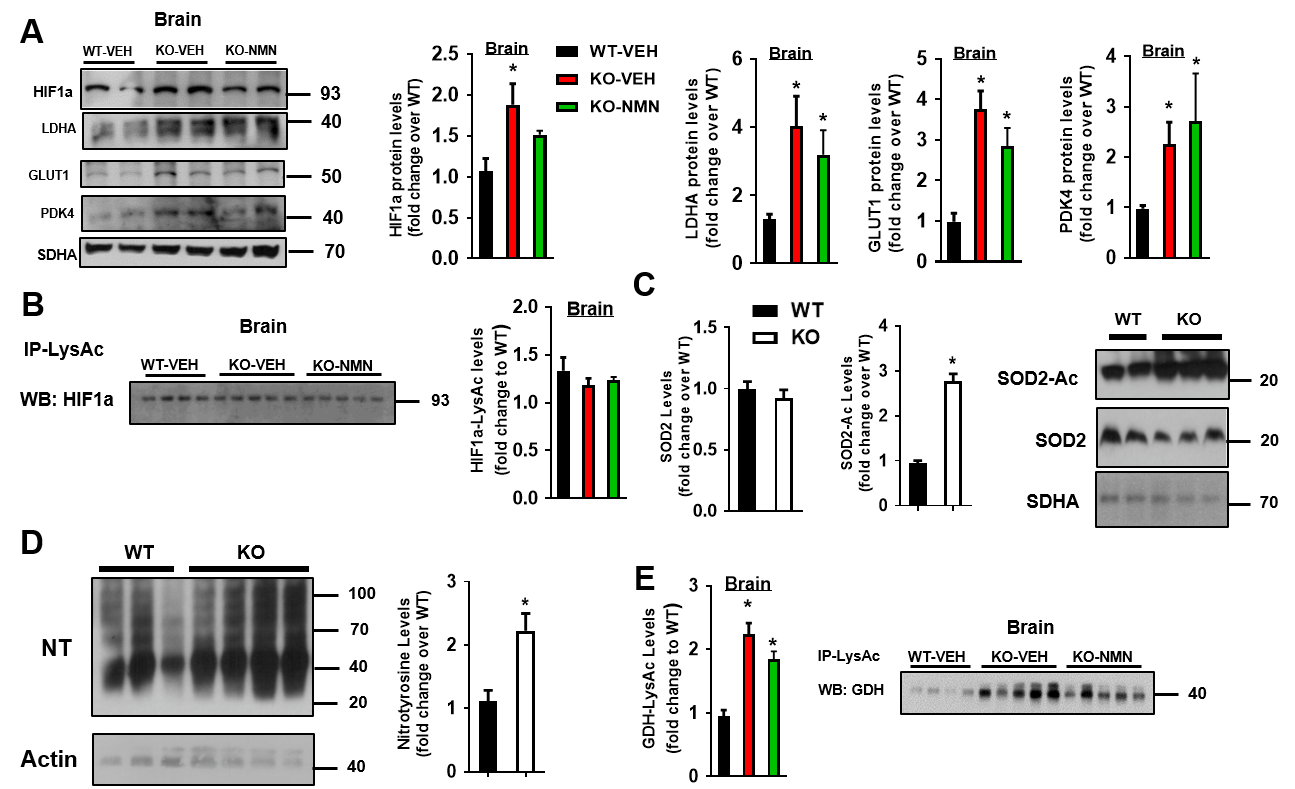


Supplementary figure 5. (A) Representative Western blot analysis of hypoxic signaling/glycolytic proteins in brain tissues of indicated mice. Quantification of HIF1a, LDHA, GLUT1 and PDK4 Western blots. SDHA was used as loading control. (B) Representative Western blots and quantification of acetylation levels of HIF1a in brains of indicated mice. Levels of (C) SOD2 protein, SOD2 acetylation (SOD2-Ac) and (D) protein nitrotyrosine (NT) from brain tissues of WT and KO mice were measured by Western blots. SDHA and actin were used as loading control. (E) GDH acetylation levels were quantified in brain tissue from indicated mice. Representative Western blots of GDH acetylation in brain from mice as indicated. One-way ANOVA with Newman-Keuls multiple comparison test and unpaired 2-tailed t-tests were used when appropriate.


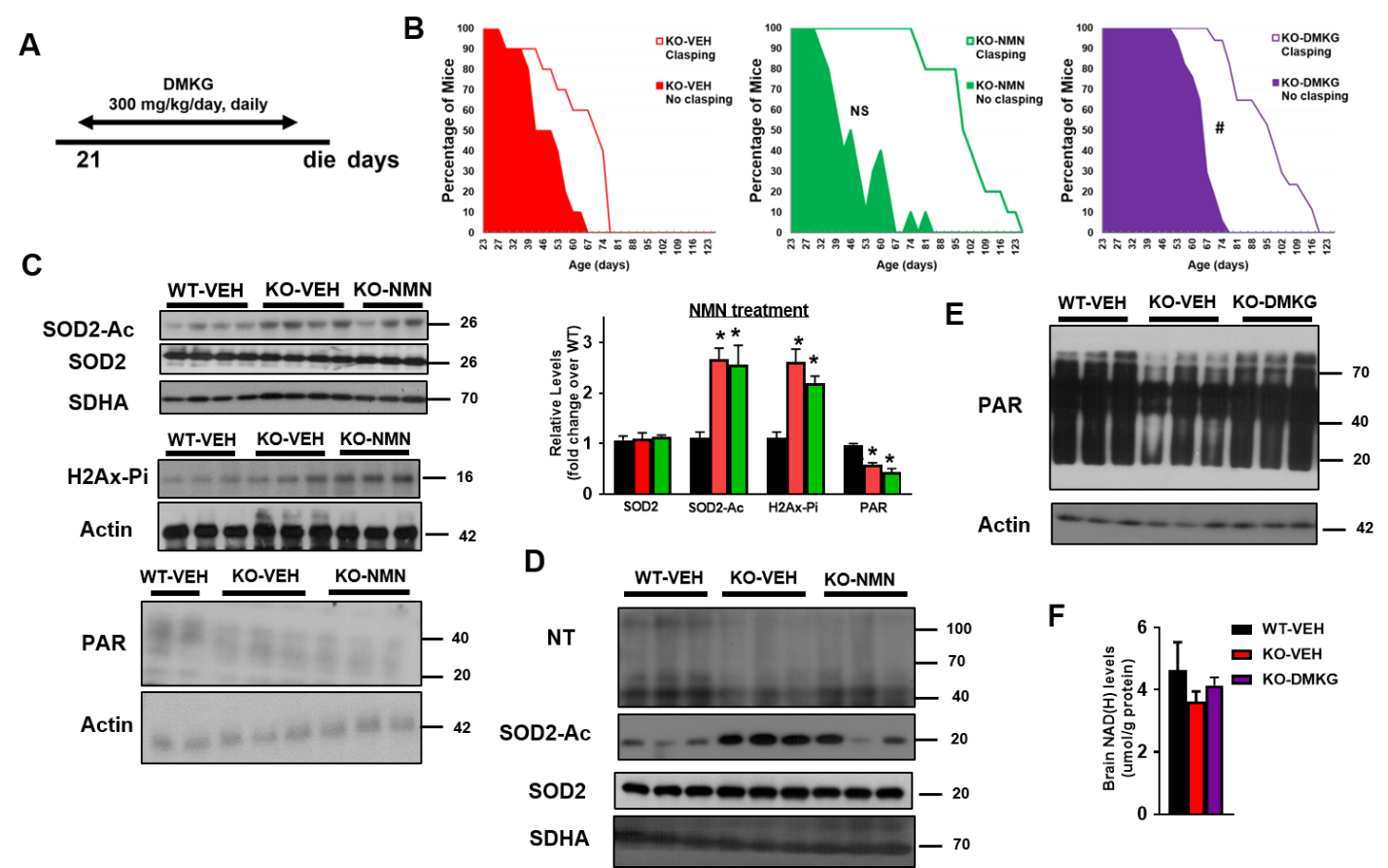
Supplementary figure 6. (A) Treatment scheme of DMKG to Ndufs4-KO mice. (B) Incidence of clasping of vehicle-, NMN-, or DMKG-treated Ndufs4-KO mice as a function of age. NS: not statistically significant versus KO-VEH. #: P<0.05 to KO-VEH. Log-rank test was used. (C) Western blots and quantification of SOD2, SOD2-Ac, H2Ax and PAR from brain tissues from the NMN-treatment cohort. (D) Levels of protein nitrotyrosine (NT), SOD2 acetylation, SOD2 protein and SDHA protein in skeletal muscle of indicated mice were measured by Western blot. (E) PAR levels from brain of indicated mice were measured. (F) NAD^+^ pool of skeletal muscle from indicated mice were measured. SDHA and actin were used as loading controls.


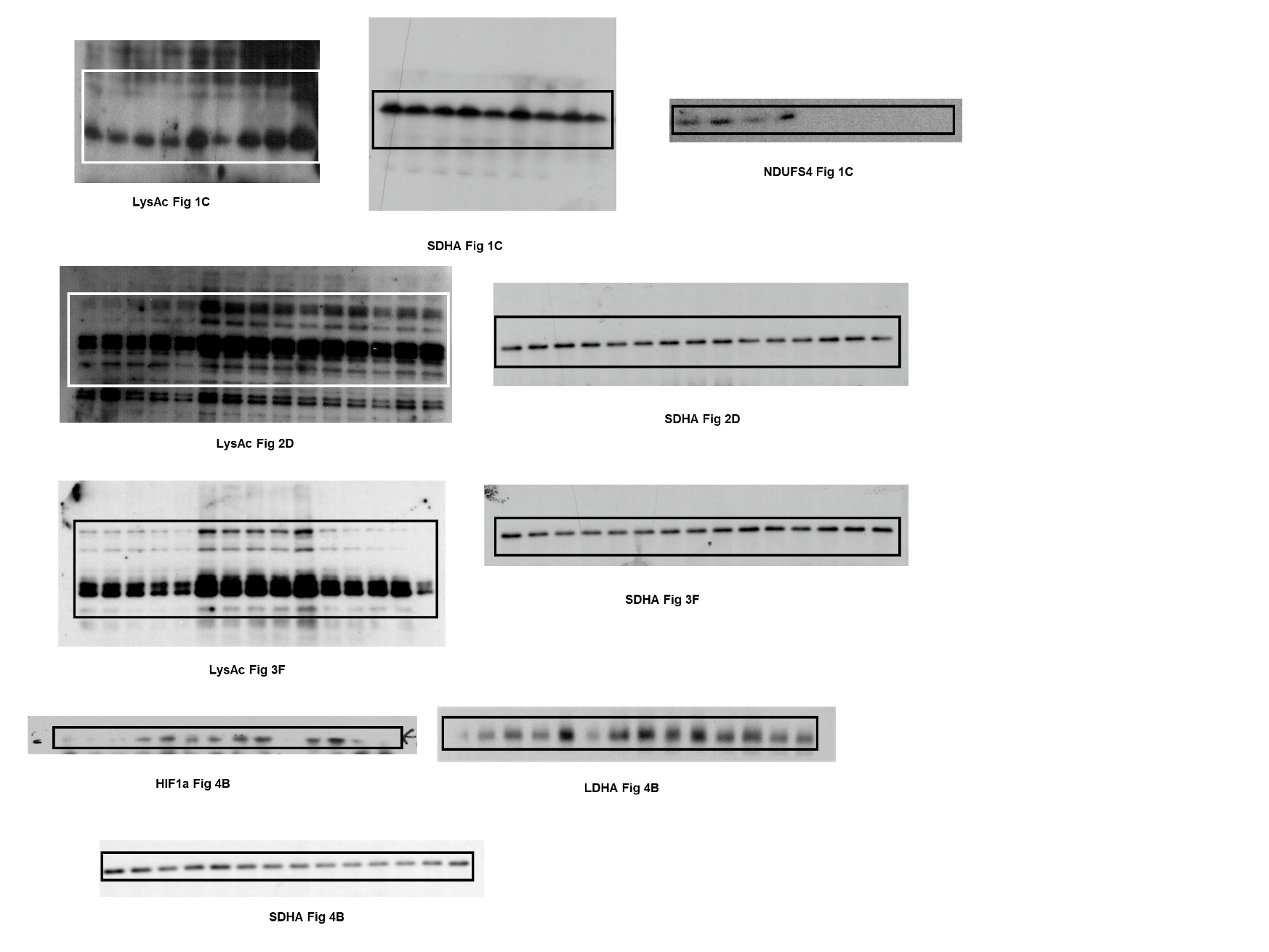


Supplementary figure 7. Full blots for main text figures.


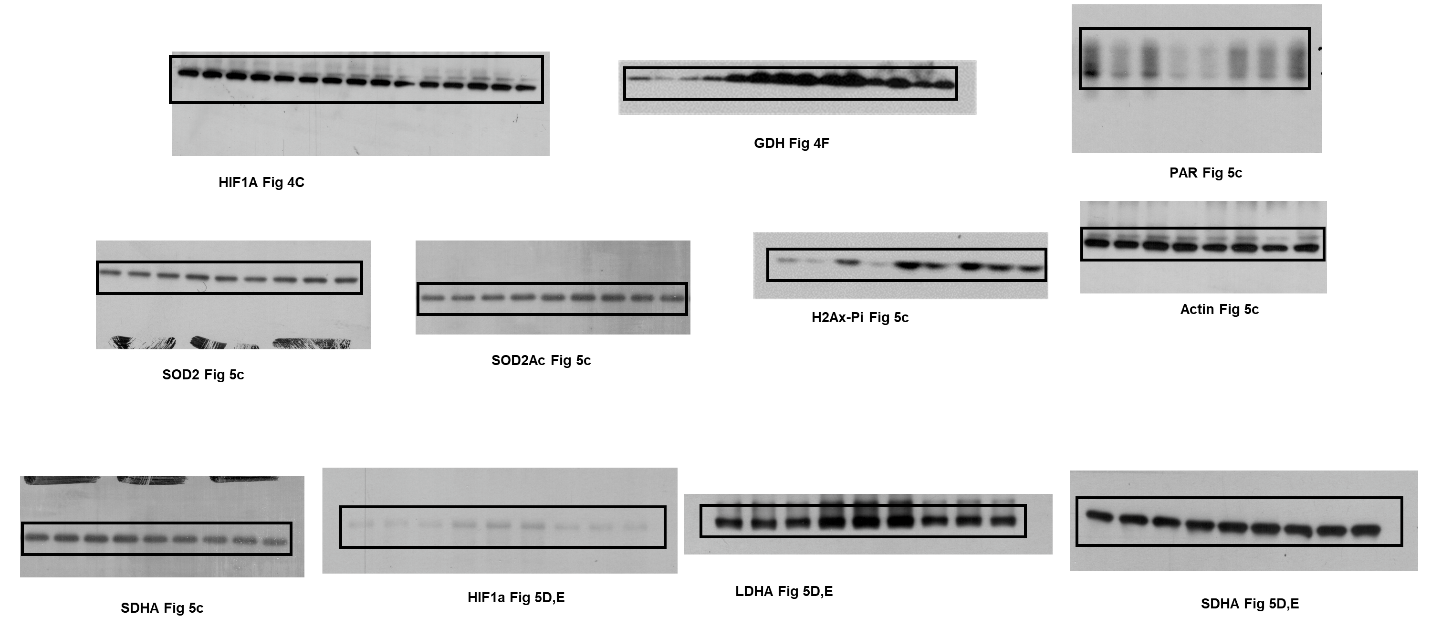


Continued Supplementary figure 7. Full blots for main text figures.
